# Photocatalytic Oxidation of Alcohols with Organic Dyes: From Homogeneous Systems to Sensitized Materials

**DOI:** 10.1002/chem.202501124

**Published:** 2025-06-02

**Authors:** Elena Tacchi, Greta Rossi, Andrea Sartorel

**Affiliations:** ^1^ Department of Chemical Sciences University of Padova Via Francesco Marzolo 1 Padova 35131 Italy

**Keywords:** alcohol oxidation, glycerol, organic dyes, photocatalysis, visible light

## Abstract

The oxidation of alcohols retains an enormous importance in industry and in synthetic organic chemistry. The renaissance in photocatalysis is offering new routes to conduct this class of transformations under benign and sustainable conditions. In this concept article, we highlight the efforts that have been conducted in light‐driven oxidation of alcohols using organic photocatalysts (PC). After an overview of relevant industrial processes, we will discuss relevant examples of PC employed in solution or when integrated in photosynthetic schemes within semiconductor slides or nanoparticles. The combination of the photochemical systems with electrochemical routes or their engineering in flow will be considered. Where possible, we highlight also mechanistic aspects that reveal the key steps involved and contribute to improve the performance of the process. We finally present an outlook on the future perspectives and developments in the field.

## Graphical abstract

We discuss the developments in the light‐driven oxidation of alcohols with PC. We report cases where the homogeneous systems can be interfaced with electrochemical tools or adapted in flow and where the PC are integrated in sensitized materials.

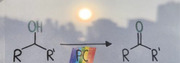

## 1 Introduction

Oxidation of alcohols retains a fundamental interest in organic and industrial chemistry.^[^ ^1^, ^2^ ^]^ The dehydrogenation of alcohols (a formal loss of 2 e^–––^ /2 H^+^) leads to carbonyl compounds as the primary oxidation products, constituting a key reaction in organic chemistry.^[^ ^3^ ^]^ Thus, the formation of carbonyl compounds by oxidation of alcohols offers chemists the easiest and most direct route, making this oxidation a popular and innovative strategy to meet the growing needs of organic synthesis in the environmental field.

Carbonyls constitute indeed one of the most important functionalities in pharmaceuticals, birthing the metaphor “carbonyl compounds are virtually the backbone of organic synthesis.”^[^ ^4^ ^]^

**Scheme 1:**
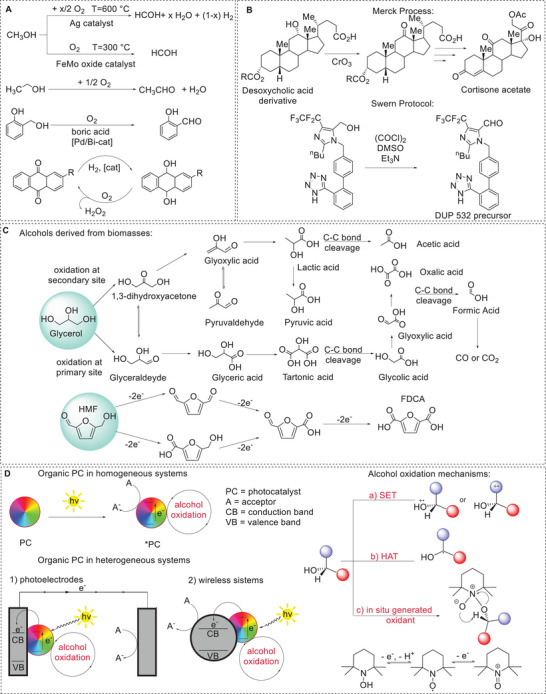
A, B) Selected examples of industrial processes for alcohol oxidation. C) Examples of alcohols derived from biomass and providing high added value oxidation products. D) Presentation of this concept article, with PC employed in homogeneous systems, in photoelectrodes, or in wireless photocatalytic nanoparticles. Representation of possible mechanistic scenarios for alcohol oxidation.

In industry, the most relevant example is oxidation of methanol to formaldehyde (about 20 Mton/year), for which different processes have been developed,^[^ ^1^, ^5^ ^]^ taking advantage of oxygen as the primary oxidant, but operating in the presence of silver or iron/molybdenum oxide catalysts at high temperatures (300–600 °C), Scheme 1A. The analogous oxidation of ethanol to acetaldehyde is less developed, but it is attracting renewed interest given the possibility of utilizing large feedstocks of bioethanol as an alternative route for acetaldehyde preparation through the Wacker oxidation of ethylene.

Oxidation of benzyl alcohols to aromatic carbonyl compounds retains interest in fragrance production, alimentary, and pharmaceutical industry. Besides benzaldehyde (70 ktons/year, produced by aerobic oxidation of toluene), an example is salicyl aldehyde (estimated 3 ktons/year^[^ ^1^ ^]^), in part produced via oxidation of salicyl alcohol (saligenin) using a Bi‐modified Pt catalyst with boric acid additive. Another example of alcohol‐to‐carbonyl conversion of industrial relevance is the anthraquinone process for the production of hydrogen peroxide (the global consumption in 2018 was about 6.5 million metric tons, and the value has been increasing rapidly since then, with a domestic market share of more than 50%^[^ ^6^ ^]^), where aerobic oxidation of hydroquinone to anthraquinone is accompanied by the formation of H_2_O_2._ The 2‐alkylanthraquinone is first hydrogenated in a catalyzed process to the corresponding hydroquinone, that in a second step reacts with O_2_ for H_2_O_2_ production without the need of a catalyst. This strategy is safer compared to those previously used because it avoids adding O_2_ and H_2_ in the same step. In addition, in the water extraction step, a rather concentrated solution (up to 40 wt % H_2_O_2_) is directly obtained without the need of expensive concentration steps.^[^ ^1^ ^]^ In the pharmaceutical industry (Scheme 1B), a well‐known process is the conversion of desoxycholic acid to cortisone acetate developed by Merck in the 1950s and 1960s.^[^ ^7^ ^]^ This oxidation‐rich method involved 11 separate oxidation steps, including the use of CrO_3_ to oxidize desoxycholic acid and access the 12‐keto derivative. Similarly, the Swern oxidation, a method for oxidizing primary or secondary alcohols to aldehydes or ketones, respectively, combines oxalyl chloride and dimethyl sulfoxide, followed by triethylamine, and has been widely used in the pharmaceutical industry, as in the case of DUP 532 derivatives.^[^ ^2^ ^]^

Oxidation of alcohols is becoming relevant also to target the conversion of cheap and available raw materials, derived from biomass (Scheme 1C). One example is glycerol^[^ ^8^ ^]^ as a by‐product of biodiesel production, for which the carbonyl products deriving from its primary oxidation are 1,3‐dihydroxyacetone and glyceraldehyde. 1,3‐dihydroxyacetone retains interest in cosmetic industry as a self‐tanning agent, but is also used as a monomer in polymer biomaterials.^[^ ^9^ ^]^ Nowadays, the only industrial‐scaled route to produce 1,3‐dihydroxyacetone is through microbial fermentation of glycerol, with many drawbacks like low productivity and a long fermentation time. So, the selective oxidation of glycerol to 1,3‐dihydroxyacetone is a very attractive alternative for industrial applications.^[^ ^10^ ^]^ Glyceraldehyde is also a constituent in cosmetic and pharmaceutical products and is a starting material in the synthesis of serin.^[^ ^9^ ^]^

Glycerol is an example of an alcohol substrate for which the overoxidation to carboxylic acid derivatives is also retaining interest. Indeed, overoxidation of glycerol leads to other diverse value‐added chemicals, such as glyceric acid, lactic acid, glycolic acid, formic acid, and carbon monoxide. A similar scenario is also found for 5‐(hydroxymethyl)furfural (HMF) – another alcohol derived from biomass – for which the oxidation targets 2,5‐furandicarboxylic acid (FDCA), a compound of interest in polymer industry.^[^ ^11^, ^12^ ^]^

Traditionally, the oxidation of alcohols in organic chemistry is accomplished by using stoichiometric amounts of chemical oxidants, such as Mn(VII) or Cr(VI) derivates,^[^ ^1^ ^]^ activate dimethylsulfoxide,^[^ ^13^ ^]^ hypervalent iodine reagents,^[^ ^14^ ^]^ creating large volumes of waste, or requiring the use of metal‐based complexes^[^ ^15^ ^]^ or nanoparticles^[^ ^16^, ^17^ ^]^ as catalysts.

The renaissance of photocatalysis allows to develop the oxidation of alcohols through the use of visible light and exploiting air or dioxygen as the primary oxidant, thus permitting designing the process in more sustainable and green conditions (Scheme 1D). Moreover, unlike traditional industrial oxidation methods, overoxidation—when undesired—can be prevented in photocatalytic processes.^[^ ^18^ ^]^ In particular, the use of organic photocatalysts (PC) enables to exploit the unique reactivity of photoexcited *PC, taking advantage of the molecular design and tunability of the organic scaffold to control light‐harvesting capability, redox potentials, and lifetime.

Recent advances in photocatalytic oxidation of alcohols via the use of organic dyes are reported in this concept; we will discuss the selected examples by focusing on the type of technology considered and on the mechanistic analysis. We mean to highlight the rational design of photocatalytic strategies rather than to provide performance indicators.

Concerning the type of technology, we will start with examples of reporting systems in homogeneous conditions, considering also the utilization of electrochemical techniques. We will then switch to photoelectrochemistry systems, taking advantage of photoanodes; this is indeed a technology that has faced a broad diffusion in the last years, in particular for water splitting and artificial photosynthesis applications. We will finally discuss wireless photocatalytic materials as a most viable technology for conducting redox transformation, taking advantage of the pros of heterogeneous catalysis among which the recovery and recyclability of the photocatalyst is the most obvious.

The mechanistic scenario should also be evaluated along transformation of the alcohol substrate, and typically three scenarios are considered: (i) a single electron transfer (SET)^[^ ^19^ ^]^ from the alcohol to the excited *PC (pathway a in Scheme 1D, producing the radical cation of the alcohol and the reduced PC^•‐^, i.e., reductive quenching of *PC); this pathway requires a suitable oxidizing power of *PC (i.e., suitable redox potential of the *PC/PC^·‐^ couple), and is especially considered for benzylic or aromatic alcohols. (ii) A hydrogen atom transfer (HAT) mechanism, where the excited‐state photocatalyst (*PC) abstracts a hydrogen atom from the alcohol substrate (pathway b in Scheme 1D).^[^ ^19^ ^]^ In this case the key reactivity parameter is the bond dissociation free energy (BDFE) of the bonds that are formed/broken; this reactivity exploits indeed the weak C─H bonds in alpha to the oxygen heteroatom in the alcohol substrate, due to the hyperconjugative effect.^[^ ^20^, ^21^ ^]^ This pathway requires the presence in the PC scaffold of a group able to bind the hydrogen atom, as in the case of quinone compounds. (iii) In situ generated oxidant, where the excited PC can finally promote a charge separation state, with the electron vacancies that are exploited to feed suitable catalysts, typically aminoxyl radical or TEMPO‐like radicals (TEMPO = 2,2,6,6‐tetramethyl‐1‐piperidinyloxy free radical^[^ ^22^, ^23^ ^]^); these then operate through a polar, 2‐electron mechanism with the oxoammonium being the active species (Pathway c in Scheme 1D).^[^ ^22^, ^23^, ^24^, ^25^, ^26^ ^]^ Modifications of aliphatic aminoxyl radicals offer the advantage of tuning the oxidation potential of the (N═O)^+^/(N─O·) couple up to more than 0.7 V (from 0.6 to 1.3 V vs. NHE),^[^ ^26^ ^]^ thus allowing them to properly design their coupling with photocatalysts’ potentials.^[^ ^26^ ^]^ Photochemical generation of reactive oxygen species (singlet oxygen ^1^O_2_ or superoxide anion O_2_^•‐^) is an additional route, but it is typically associated with low selectivity. In particular, the superoxide anion may lead to autooxidation chain pathways.

## 2 PC in Homogeneous Phase

The oxidation of alcohols with PC operating under visible light irradiation is an emerging field that is attracting growing interest. Hence, many efforts have been made to select/develop/optimize the organic photocatalyst, and several examples, including Eosin Y,^[^ ^27^ ^]^ Rose Bengal,^[^ ^28^ ^]^ thioxanthone,^[^ ^3^ ^]^ 1,2,3,5‐Tetrakis(carbazol‐9‐yl)‐4,6‐dicyanobenzene (4CzIPN)^[^ ^29^, ^30^ ^]^, and quinones^[^ ^31^, ^32^, ^33^, ^34^, ^35^, ^36^ ^]^ were reported.

Das et al. developed the photocatalytic oxidation of alcohols under visible light irradiation based on the very cheap 9‐flourenone (112 €/kg). The system showed a broad substrate scope for aliphatic, heteroaromatic, aromatic, and alicyclic alcohols, which were converted to the corresponding carbonyl compounds in good yield (24 examples, up to > 99% yield, 33 turnovers), and included also the oxidation of steroids, in particular for the preparation of terephthalaldehyde and androstenedione. The reactions were conducted under an air atmosphere by irradiation with a blue light‐emitting diode (LED) in DMSO as a solvent (a decrease in the yield was observed when > 3% v/v water was present).^[^ ^34^ ^]^

As an aromatic ketone, fluorenone is characterized by a good intersystem crossing yield, that allows light‐induced population of the triplet excited state,^[^ ^37^ ^]^ being a powerful oxidant.

Similarly, quinones as aromatic dicarbonyl compounds, are characterized by highly oxidant triplet excited states, possibly engaging SET and HAT steps in photocatalytic oxidation processes,^[^ ^33^, ^38^ ^]^ also embedded in metal‐organic frameworks.^[^ ^39^ ^]^ Photoexcited quinones exhibit an O‐centered radical character, closely resembling electrophilic alkoxyl radicals and displaying exceptional hydrogen abstraction capabilities, thereby enabling the cleavage of C─H bonds of the target substrate. Moreover, quinones show the ability to undergo a two‐electron and two‐proton reduction to hydroquinones, that can react with oxygen to produce hydrogen peroxide. The ability of quinones to photooxidize alcohols has been long recognized.^[^ ^40^, ^41^, ^42^ ^]^ Meng et al. reported a strategy for the homogeneous visible light photocatalytic preparation of H_2_O_2_ by oxidation of isopropanol or other lower alcohols using commercially available 2‐ethylanthraquinone (EAQ) as the photocatalyst and operating through HAT. The system worked under room temperature conditions with irradiation provided by a 427 nm blue LED and stirring under 0.5 MPa O_2_ (productivity of H_2_O_2_ up to 323 mM·h^−1^) and was transferrable to flow conditions, that enabled to improve the productivity of ca. two orders of magnitude.^[^ ^31^ ^]^ A proper design of the flow photoreactor is an important target to boost the photocatalysis performance.^[^ ^43^ ^]^ Helaja et al. used quinones embedding electron‐withdrawing groups to access the photochemical oxidation of nonactivated secondary aliphatic alcohols (29 examples, up to 99% NMR yield, 10 turnovers based on the quinone).^[^ ^33^ ^]^ In many cases targeting photochemical H_2_O_2_ production from the reduction of O_2_,^[^ ^44^ ^]^ the concomitant alcohol oxidation process is less investigated, and the substrate being oxidized is referred simply as hydrogen atom donor. Of course, developing an appealing alcohol oxidative process in tandem to H_2_O_2_ production represents a win/win possibility.

Recently, in our group, we reported the photooxidation of glycerol into formic acid, occurring in water with O_2_ as the oxidant after 22 hours and without the need for additives, by irradiation with a 415 nm LED of a 9,10‐anthraquinone‐2,6‐disulphonate disodium salt (AQDS) as photocatalyst (conversion of glycerol up to 79% leads to 30% yield of formic acid, ca. 15 turnovers).^[^ ^36^ ^]^

**Scheme 2:**
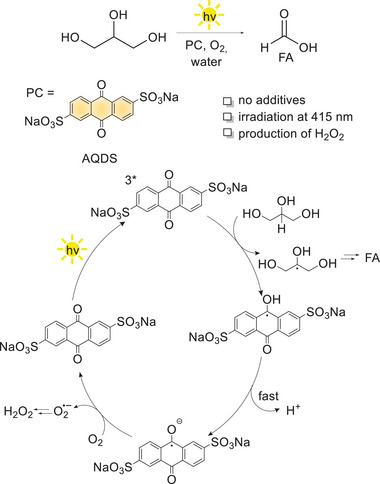
Photochemical oxidation of glycerol to formic acid with AQDS photocatalyst and reaction mechanism. Adapted from ref. [36] with permission from Wiley.

Investigation of the photochemical mechanism combining transient absorption and EPR spectroscopies supports an initial hydrogen atom abstraction (HAT) from the secondary position of glycerol substrate operated by the triplet excited state ^3*^AQDS of the photocatalyst (estimated BDFE of 112 kcalmol^−1^). This step leads to the formation of dihydroxyacetone as the primary oxidation product (further oxidized to formic acid), and to the reduced forms of the AQDS photocatalysts that are responsible for H_2_O_2_ production through reduction of dioxygen (Scheme 2).

## 3 Combination of Electro‐ and Photochemistry

Another promising approach that has recently been investigated for homogeneous alcohol oxidation is the combination of photochemical and electrochemical activation, that can follow different strategies.^[^ ^45^ ^]^

**Scheme 3:**
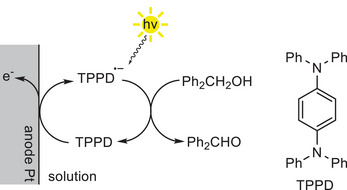
Electro/photochemical activation of TPPD for oxidation of benzyl alcohol. Adapted from ref. [46] with permission from the Royal Society of Chemistry.

The first example of this approach for alcohol oxidation was reported in 1982 by Moutet and Reverdy.^[^ ^46^ ^]^ In their study, N,N,N',N'‐tetraphenyl‐*p*‐phenylenediamine (TPPD) was used for electro/photochemical benzylic alcohol oxidation to benzaldehyde. The TPPD was initially electrochemically oxidized at the anode to its radical cation TPPD^•+^, that was subsequently photochemically excited (*λ* >360 nm), generating the excited radical cation *TPPD^•+^ as a super‐oxidant (Scheme 3). *TPPD^•+^ was postulated to be the acceptor of a SET from the benzyl alcohol, thus initiating the oxidation pathway.^[^ ^47^ ^]^ The generation of super‐oxidants (i.e., excited states of electrochemically oxidized species) is facing an increase of interest in the last few years, since this allows the expansion of the reactivity of organic compounds beyond the traditional redox window.^[^ ^47^, ^48^, ^49^, ^50^ ^]^

A different approach that combines photonic and electronic activation of the catalyst in oxidation reactions is the use of anodic potential in substitution of a sacrificial electron acceptor (including O_2_) to regenerate the photocatalyst.

**Scheme 4:**
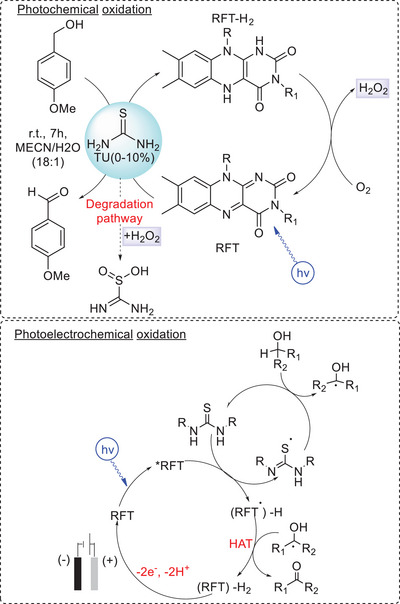
Photochemical^[^ ^51^ ^]^ top) and photoelectrochemical^[^ ^53^ ^]^ bottom) oxidation of alcohols with RFT photocatalyst, mediated by thiourea. Optimized photoelectrochemical conditions: RFT (5 mol %), 1,3‐diisopropylthiourea (10 mol %), LiOTf (0.1 mM in 3.5 mL CH_3_CN), H_2_O (0.2 mL), cell voltage *U*_cell_ = 2.5 V (initial anodic potential *E*_anode_ = 0.8 V vs. SCE), blue LED.

The example of riboflavin tetraacetate (RFT) photocatalyst clarifies how this strategy could be very useful for alcohol oxidation. In 2008, Köenig reported the thiourea‐mediated photoxidation of 4‐methoxybenzyl alcohol using RFT photocatalyst and O_2_ as the terminal oxidant. The addition of thiourea, as co‐catalyst or covalently bonded to the riboflavin skeleton, resulted in a 30‐fold increase in quantum yield up to 1.8% and turnovers up to 580.^[^ ^51^ ^]^ Mechanistic investigation reveals that thiourea acts as an efficient electron mediator between the photocatalyst and the substrate (Scheme 4), while O_2_ reduction leads to the production of hydrogen peroxide (this route was exploited for further H_2_O_2_ activation, in a dual catalysis approach^[^ ^52^ ^]^). This strategy was proven successful for activating benzyl alcohol but was less effective for more demanding substrates such as aliphatic alcohols.^[^ ^47^ ^]^ Lin et al.^[^ ^53^ ^]^ were able to extend the oxidation to more challenging aliphatic alcohols through a photoelectrocatalytic strategy. Their study revealed that the main limitation of Köenig's approach was the presence of O_2_ as terminal oxidant. Indeed, the regeneration of RFT via O_2_ reduction leads to the formation of H_2_O_2_, which degrades thiourea. This decomposition pathway outcompetes the substrate oxidation for challenging substrates such as 4‐*tert*‐butylcyclohexanol (*E* > 1.9 vs. SCE), leading to only a 3% yield of ketone. Replacing the O_2_ terminal oxidant with carbon foam anode enables the unlocking of new mechanistic pathways, making possible the oxidation of primary and secondary aliphatic alcohols via HAT mediated by the thiourea‐based radical that, thanks to this alternative approach that excludes O_2_ as terminal oxidant, doesn't degrade during catalysis (Scheme 4). In the optimized conditions, the authors reached a nearly quantitative conversion of 4‐*tert*‐butylcyclohexanol with very selective formation of the corresponding ketone (95% ^1^H‐NMR yield, 91% isolated yield). The oxidation of other secondary alcohols and of unactivated primary alcohols (eight examples) with high isolated yield of ketones (53‐90%, ca. 20 turnovers) was also reported in the same work. These examples highlight the relevance of combining the thiourea mediator with the photoelectrochemical approach, allowing the broadening of oxidation's scope without requiring a more oxidizing exited state potential. The electrochemical regeneration of the photocatalyst was also recently considered by Ravelli and colleagues for the photochemical oxidation of alcohols mediated by the inorganic decatungstate TBA_4_W_10_O_32_ (TBA = tetrabutylammonium).^[^ ^54^ ^]^

In addition to the photoelectrochemical approach, Cibulka et al. were able to access the oxidation of challenging alcohol derivatives such as 4‐trifluoromethyl alcohol employing a CF_3_‐substituted isoalloxazine, in the presence of Cs_2_CO_3_.^[^ ^55^ ^]^ In this example, the extension of reaction scope is reached thanks to the higher excited state potential of the functionalized isoalloxazine with respect to the RFT (2.67 V vs. 1.67 V vs. SCE, respectively), allowing to develop the photochemical oxidation in the absence of the thiourea mediator (three examples, up to 87% isolated yield, 17 turnovers).

## 4 Dye Sensitized Photoelectrodes

PC can be properly integrated to semiconductive slides through anchoring groups,^[^ ^56^ ^]^ for the fabrication of dye sensitized photoelectrodes as the constituents of photoelectrochemical cells. This technology takes inspiration from the dye‐sensitized solar cells (DSSC) for converting solar light into electricity,^[^ ^57^ ^]^ with the additional complexity of conducting chemical redox reactions.^[^ ^58^ ^]^ In particular, in a sensitized photoanode, light is absorbed by the PC, and the excited electron is transferred into the conduction band of the semiconductor; this is often referred to as photoinduced electron injection and may occur in a timescale ranging from hundreds of femtoseconds to picoseconds.^[^ ^59^ ^]^ The primary result of photoinduced electron injection is a charge separation, the pillar of natural and artificial photosynthetic processes. While the injected electron is conveyed to the cathode through the external circuit (eventually with the application of a bias) where a reduction reaction takes place, the electron vacancy is exploited for an oxidation process, and oxidation of water was mainly targeted to develop water splitting devices.^[^ ^60^ ^]^ More recently, the investigation of anodic organic processes was considered, and dye‐sensitized photoanodes for C─H activation^[^ ^61^ ^]^ and for alcohol oxidation^[^ ^62^ ^]^ were developed. The PC considered for integration in photoanodes took advantage of previous investigations in the field of DSSC and belongs to the family of porphyrinoid derivatives, thienopyrroledione‐based organic dye,^[^ ^63^, ^64^ ^]^ diketopyrrolopyrrole dyes,^[^ ^65^ ^]^ perylene,^[^ ^66^ ^]^ and polyquinoids.^[^ ^67^ ^]^ These studies target mainly photoelectrocatalytic benzyl alcohol dehydrogenation, operating in organic solvents and employing aminoxyl radical co‐catalysts and a base.

**Scheme 5:**
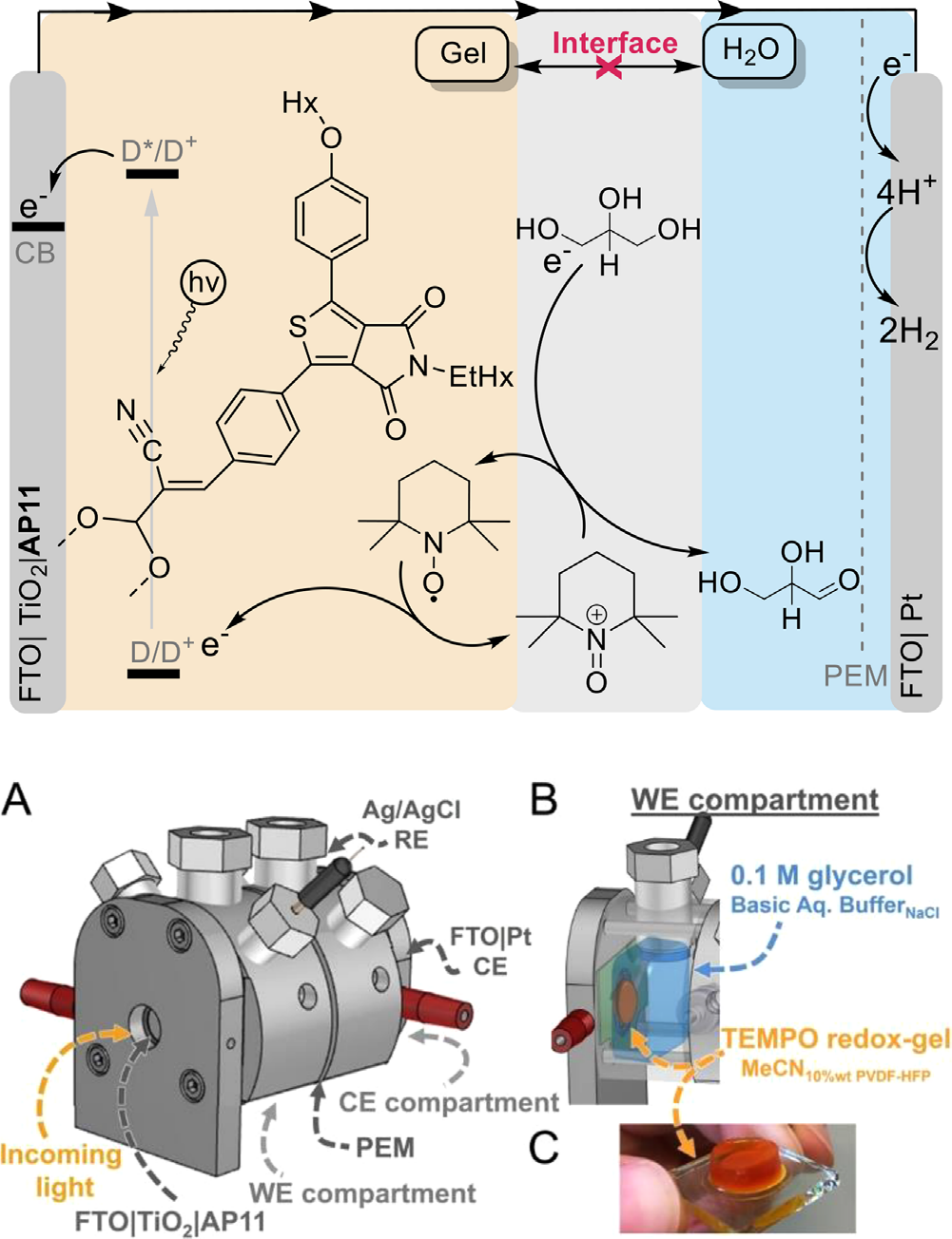
Top: schematic view of the photoanode based on a thienopyrroledione‐based dye (AP11) protected by an organogel for photoelectrochemical oxidation of glycerol to glyceraldehyde coupled to cathodic H_2_ evolution. Bottom, panels A‐C: representation of the experimental set‐up and image of the photoanode. Reprinted from ref. [68] with permission from Wiley.

An interesting innovation was recently reported by Reek and co‐workers, who developed a photoelectrode capable of performing photoelectrochemical oxidation of glycerol to glyceraldehyde, in aqueous solution, while producing H_2_ from proton reduction at the cathode (Scheme 5).^[^ ^68^ ^]^ The key component is a thienopyrroledione‐based dye (AP11), encased in a few mm thick organogel layer, based on acetonitrile‐poly(vinylidene fluoride‐co‐hexafluoropropylene), PVDF‐HFP, gel electrolytes. The function of the gel soft material is dual, protecting the surface of the photoanode from direct contact with the aqueous electrolyte, and mediating the transfer of the TEMPO catalyst from the electrode surface to the aqueous phase. The presence of the gel was beneficial in the performance of the photoanode, providing an increased stability toward dye detachment. A photocurrent density around 200 µA·cm^−2^ was registered in mildly basic aqueous medium, remaining stable for up to 2 days and being associated with selective oxidation of glycerol to glyceraldehyde with a quantitative Faradaic efficiency.

It is worth commenting that to date, dye‐sensitized photoanodes for alcohol oxidation are characterized by photocurrent densities of few hundred of µA·cm^−2^ (a photocurrent density of 100 µA·cm^−2^ corresponds to an alcohol oxidation rate of 1.9 µmol·h^−1^·cm^−2^, assuming a 2‐electron process and a 100% Faradaic efficiency), and are thus far from real applicability. Improvement of photocurrent should consider efficient light absorption, electron injection while retarding unproductive charge recombination. Strategies to improve these aspects are the nanostructuring of the semiconductor, the use of redox mediators, and the adoption of insulating layers in the semiconductor.^[^ ^69^, ^70^ ^]^

However, photoelectrochemical cell scale‐up would still require the use of an external circuit. In this sense, the use of a wireless photocatalytic material, capable of simultaneously conducting the two redox semi‐reactions is highly desirable. Indeed, already in 2014, heterogeneous wireless photocatalysts (or mixed colloid photocatalysts) were considered by H.B. Gray and co‐workers as the most sustainable and scalable technology for large‐scale applications of photochemical water splitting,^[^ ^60^ ^]^ although at that time the technology was considered not mature. In the following years, significant advancement in the field has been reached.^[^ ^71^ ^]^ Concerning alcohol oxidation, significant reports on such types of materials, in the form of dye‐sensitized semiconductive nanoparticles, are discussed in the following paragraph.

## 5 Wireless Sensitized Nanoparticles

**Scheme 6:**
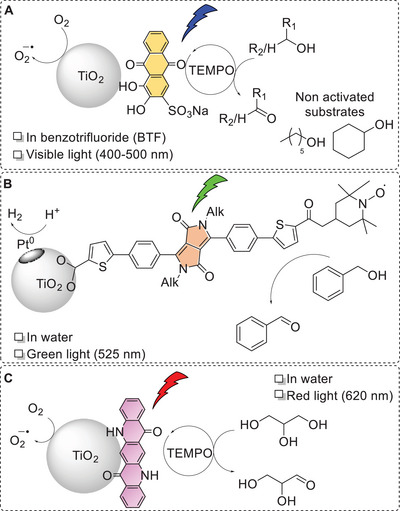
Photochemical oxidation of alcohols with dye‐sensitized semiconductor nanoparticles. A: Work by Ma and Zhao using ARS sensitized TiO_2_.^[^ ^72^ ^]^ B: Work by Vauthey, Coutsolelos, and Odobel on diketopyrrolopyrrole‐sensitized TiO_2_, embedding Pt and aminoxyl radical catalytic sites for hydrogen evolution and alcohol oxidation, respectively.^[^ ^74^ ^]^ C: Our work on quinacridone‐sensitized TiO_2_ nanoparticles for photochemical oxidation of glycerol to glyceraldehyde in water with red light (620 nm).^[^ ^75^ ^]^

The operating principles of dye‐sensitized semiconductive nanoparticles are similar to those of dye‐sensitized photoelectrodes: photoinduced charge separation via light absorption by the PC and electron injection into the conduction band of the semiconductor, and promotion of the two redox semi‐reactions at the surface of the material, possibly taking advantage of co‐catalysts. A seminal contribution in the application of this kind of material in photochemical alcohol oxidation was reported by Ma, Zhao, and co‐workers, who sensitized TiO_2_ nanoparticles with Alizarin Red S (ARS) dye (Scheme 6, panel A). When coupled to a homogeneous TEMPO co‐catalyst, the material enabled the aerobic oxidation of alcohols to carbonyl compounds in benzotrifluoride solvent and exploited 400–500 nm light; the scope of the reactivity was mainly targeting benzyl or allyl alcohols, reaching up to quantitative conversion and turnover of > 650 based on the ARS dye; the system was active also for oxidation of 1‐hexanol to hexanal and of cyclohexanol to cyclohexanone. The oxidation of alcohol was coupled to reduction of oxygen to superoxide anion O_2_^•‐^, further undergoing dismutation to hydrogen peroxide. The authors were able to trace the light‐activated mechanistic step by EPR spectroscopy.^[^ ^72^ ^]^ This work inspired further investigation on similar systems, that took advantage of other organic dyes, such as Eosin Y.^[^ ^73^ ^]^

Moreover, the versatility of these systems enables to consider alternative reduction processes coupled to alcohol oxidation. A recent example was reported by Vauthey, Coutsolelos, Odobel, and co‐workers, who developed diketopyrrolopyrrole‐sensitized TiO_2_ nanoparticles for the oxidation of benzyl alcohols coupled to hydrogen evolution, operating in water with green light (Scheme 6, panel B).^[^ ^74^ ^]^ In this system, the aminoxyl radical catalyst for alcohol oxidation was covalently integrated to the organic photocatalyst, while Pt co‐catalysts for proton reduction to hydrogen were anchored onto the TiO_2_ surface. Irradiation of the particles in water (pH 8) led to simultaneous oxidation of benzyl alcohol derivatives to the corresponding benzaldehyde and evolution of hydrogen, with up to 50 turnovers based on the organic photocatalyst. Moreover, the two products of interest are easily separable, since H_2_ evolves in the gas phase of the reaction while benzaldehyde forms in solution. While a 1:1 stoichiometry of aldehyde and H_2_ should be ideally expected (both redox semi‐reactions involve 2 e^−^), a slight excess of H_2_ with respect to the aldehyde was observed, being attributed to the more efficient Pt catalyzed process with respect to the aminoxyl‐based one. However, this discrepancy may suggest either the occurrence of a competitive oxidative process or accumulation of oxidative equivalents at the photocatalyst.

In our group, we have recently reported TiO_2_ nanoparticles sensitized with a quinacridone pigment for the photochemical oxidation of glycerol to glyceraldehyde in water (47.5±5.0 µmol∙g_NP_^−1^∙h^−1^ of productivity and ca. 80% selectivity) and exploiting red light (up to 620 nm) in the presence of TEMPO co‐catalyst, Scheme 6 panel C.^[^ ^75^ ^]^ Besides the easiness of synthesis of the material that does not require installation of an anchoring group in the pigment, the exploitation of red light is reached thanks to the aggregation state of the pigment. The material shows also recyclability, reaching up to 20 turnovers based on quinacridone.

In conclusion of this section, the flexibility of the dye‐sensitized nanoparticles approach allows to consider future developments by modulating the reductive reactivity, for instance, targeting carbon dioxide reduction, for which sensitized nanoparticles have been already developed.^[^ ^76^ ^]^ Also, the development of redox‐neutral processes involving transformation of a single organic substrate may be further considered.^[^ ^77^, ^78^ ^]^

## 6 Summary and Outlook

In this concept article, we discussed the use of PC for the light‐driven oxidation of alcohols. We reported cases where the homogeneous systems can be interfaced with electrochemical tools or adapted in flow, and where the PC are integrated in sensitized materials. Homogeneous photocatalysis is operationally simple; typically, it exploits PC in few percent with respect to the substrate, but of course it faces the disadvantage of photocatalyst recovery. The combination with electrochemistry is an elegant solution to boost the photocatalysis, for example, prolonging the stability of the PC through its electrochemical regeneration or expanding the operating potential through the super‐oxidant approach. This comes at the expense of an increase in complexity of the experimental setup, that should consider also a proper design of the cell. The integration of organic PC into materials, either photoelectrodes or sensitized nanoparticles, offers the possibility to exploit a photoinduced charge‐separated state and combine oxidation of alcohols to a valuable reduction process, while providing the advantages of heterogeneous catalysis. As discussed in the previous sections, photoelectrodes require the use of an external circuit, while wireless photocatalytic materials appear to be the suitable technological solution for scalability purposes.

We expect that further development in the field should target the following aspects: (i) *photocatalyst*: tuning the desired properties of the photocatalyst can follow a proper molecular design;^[^ ^79^ ^]^ nevertheless, the use of commercially available, cheap, and nontoxic industrial pigments should also be considered for scalability purposes.^[^ ^80^ ^]^ (ii) *Reaction conditions*: improvement of the sustainability of reaction conditions, considering the use of the low‐energy portion (λ > 600 nm)^[^ ^58^ ^]^ of solar light emission and extending the use of green solvents^[^ ^81^ ^]^ to conduct the target photochemical transformations. (iii) *Target processes*: the use of largely available and cheap substrates, as glycerol and furfural derivatives previously mentioned, is expected to rapidly expand; boosting the utility of the overall process should be considered by coupling to alcohol oxidation a suitable tandem reduction route: this was already implemented in dye‐sensitized electrodes and nanoparticles by considering carbon dioxide^[^ ^65^ ^]^ or proton reduction,^[^ ^74^ ^]^ but it can be extended to reduction of organic compounds, for possible coupling with the products of the oxidative pathway. (iv) *Technology*: examples of flow technologies were discussed in this concept; however, the upgrade of the photochemical systems to large‐scale devices should consider the development of real photoreactors or photoelectrochemical cells; as an example, the use of gas diffusion electrodes in flow cells enabled the reduction of carbon dioxide to reach impressive current densities up to 150 mA·cm^−2^.^[^ ^82^ ^]^ In water‐splitting devices, in ten years, improvement in technology enabled to pass from 1 cm^2^ sized devices^[^ ^83^ ^]^ to 100 m^2^ ones, operating for more than 1 year with solar radiation.^[^ ^71^ ^]^

## Conflict of Interests

The authors declare no conflict of interest.

## Data Availability

Data sharing is not applicable to this article as no new data were created or analyzed in this study.
